# Supplementary material for: Survival study of cytoreductive prostatectomy for prostate cancer with lung metastasis: A propensity score matching study based on the SEER database
Source: PLoS One. 2026 Feb 23;21(2):e0342758. doi: 10.1371/journal.pone.0342758 (PMC12928561; doi:10.1371/journal.pone.0342758)
Supplement: Attachment — Submitted filename: Response to Reviewers.docx [file pone.0342758.s002.docx]

When submitting your revision, we need you to address these additional requirements.

1. Please ensure that your manuscript meets PLOS ONE's style requirements, including those for file naming. The PLOS ONE style templates can be found at https://journals.plos.org/plosone/s/file?id=wjVg/PLOSOne_formatting_sample_main_body.pdf and

<https://journals.plos.org/plosone/s/file?id=ba62/PLOSOne_formatting_sample_title_authors_affiliations.pdf>

Our response：Thanks to the editor's reminder, I have become familiar with the relevant requirements and have made corresponding modifications to the format of the manuscript.

2. Thank you for stating the following in the Competing Interests section:

“An-Ping Xiang designed the study and drafted and revised the manuscript, Xue-Feng Yuan and Zhen-Qian Qin analyzes the data and draw graphs.”

Please confirm that this does not alter your adherence to all PLOS ONE policies on sharing data and materials, by including the following statement: "This does not alter our adherence to  PLOS ONE policies on sharing data and materials.” (as detailed online in our guide for authors http://journals.plos.org/plosone/s/competing-interests).  If there are restrictions on sharing of data and/or materials, please state these. Please note that we cannot proceed with consideration of your article until this information has been declared.

Please include your updated Competing Interests statement in your cover letter; we will change the online submission form on your behalf.

Our response：We have updated Competing Interests statement in new version of cover letter

3. Please note that your Data Availability Statement is currently missing a direct link to access each database. If your manuscript is accepted for publication, you will be asked to provide these details on a very short timeline. We therefore suggest that you provide this information now, though we will not hold up the peer review process if you are unable.

Our response ：My manuscript has been revised and the website address of the database has been added.

4. If the reviewer comments include a recommend

dation to cite specific previously published works, please review and evaluate these publications to determine whether they are relevant and should be cited. There is no requirement to cite these works unless the editor has indicated otherwise.

Our response ：Thanks to the editor's reminder, the reviewers did not have this requirement for my article.

Reviewers' comments:

Reviewer's Responses to Questions

**Comments to the Author**

1. Is the manuscript technically sound, and do the data support the conclusions?

The manuscript must describe a technically sound piece of scientific research with data that supports the conclusions. Experiments must have been conducted rigorously, with appropriate controls, replication, and sample sizes. The conclusions must be drawn appropriately based on the data presented.

Reviewer #1: Partly

Reviewer #2: No

Reviewer #3: No

Reviewer #4: Yes

2. Has the statistical analysis been performed appropriately and rigorously?

Reviewer #1: I Don't Know

Reviewer #2: No

Reviewer #3: No

Reviewer #4: Yes

3. Have the authors made all data underlying the findings in their manuscript fully available?

The [PLOS Data policy](http://www.plosone.org/static/policies.action#sharing) requires authors to make all data underlying the findings described in their manuscript fully available without restriction, with rare exception (please refer to the Data Availability Statement in the manuscript PDF file). The data should be provided as part of the manuscript or its supporting information, or deposited to a public repository. For example, in addition to summary statistics, the data points behind means, medians and variance measures should be available. If there are restrictions on publicly sharing data—e.g. participant privacy or use of data from a third party—those must be specified.

Reviewer #1: Yes

Reviewer #2: Yes

Reviewer #3: No

Reviewer #4: Yes

4. Is the manuscript presented in an intelligible fashion and written in standard English?

PLOS ONE does not copyedit accepted manuscripts, so the language in submitted articles must be clear, correct, and unambiguous. Any typographical or grammatical errors should be corrected at revision, so please note any specific errors here.

Reviewer #1: Yes

Reviewer #2: Yes

Reviewer #3: No

Reviewer #4: Yes

5. Review Comments to the Author

Please use the space provided to explain your answers to the questions above. You may also include additional comments for the author, including concerns about dual publication, research ethics, or publication ethics. (Please upload your review as an attachment if it exceeds 20,000 characters)

Reviewer #1: This study represents a valuable contribution to the field of urologic oncology, addressing a clinically relevant question regarding the role of cytoreductive prostatectomy in prostate cancer patients with lung metastasis. The use of a large, population-based dataset from the SEER database and the application of propensity score matching to minimize selection bias are significant strengths. The manuscript is well-structured, the methodology is sound, and the conclusions are clearly supported by the data. The topic is timely and of great interest to clinicians seeking to optimize treatment strategies for metastatic prostate cancer. However, to further strengthen the manuscript, the following revisions are suggested:

1.**The final matched cohort consists of only 79 patients per group. While PSM helps balance covariates,** **such a small sample may limit the statistical power to detect clinically meaningful differences. Please discuss this limitation and consider performing a power calculation or sensitivity analysis.**

Our response：Thank you to the reviewer for your valuable suggestions! Among the large amount of data in this study, only 161 patients with prostate cancer and lung metastasis who met the research criteria underwent surgical treatment. To ensure the reliability of the research results, I conducted PSM matching on the data. When the caliper value was set at 0.7, the surgical group and the non-surgical group were 80 cases each; when the caliper value was set at 0.5, the surgical group and the non-surgical group were 79 cases each. Therefore, in order to ensure the reliability of the research data, I chose the more stringent caliper value of 0.5. Therefore, the authors believe that although the data of this study is not large, the conclusion is worthy of belief.

2.
**The term “reductive prostatectomy” is ambiguous. Please clarify whether this refers to** **radical prostatectomy, cytoreductive surgery, or palliative debulking. Specify the surgical intent and extent in the Methods section.**

Our response：Thank you to the reviewer for your valuable suggestions. I have made revisions to the relevant issues in the full text and have adopted a more appropriate term "cytoreductive prostatectomy";

**the surgical intent and exten**t：I have provided supplementary explanations in the abstract: "The purpose of palliative debulking is to reduce the tumor burden. Only the primary lesion of the prostate is removed, while metastatic lesions are not subjected to surgical resection."

3.
**Key prognostic factors such as ECOG performance status, comorbidities, PSA doubling time, and specific systemic therapies (e.g., AR-targeted therapies, chemotherapies) are not accounted for. These omissions may introduce residual confounding. Please acknowledge this limitation.**

Our response：Thank you for the valuable suggestions provided by the reviewers. It is indeed due to the lack of prognostic parameters in the SEER database that a comprehensive assessment of these prognostic factors cannot be conducted. I have explained these deficiencies in the article and further research is needed in the future.

I made the following explanations in the article.：

“In addition, the study subjects were limited to patients aged 60 to 79, excluding the younger or older population, so caution is needed when extrapolating the results to other age groups. Finally, as a retrospective observational study, despite the use of the PSM approach, it was not possible to completely eliminate the influence of residual confounding factors such as the patient's physical condition (ECOG score), tumor molecular characteristics (such as AR-V7 expression, DNA repair gene defect), complications, PSA doubling time and specific systemic therapy which may affect surgical decisions and prognosis, but were not recorded in the database.”

4.**The study does not distinguish between oligometastatic and polymetastatic disease. Patients with solitary lung metastasis may behave differently than those with multiple metastases. Subgroup analysis based on metastatic burden would strengthen the conclusions.**

Our response：We are grateful for the valuable suggestions provided by the reviewer. The SEER database stores the records of distant metastasis (including lung metastasis) in a binary classification format, such as "yes/no" in fields like “CS mets at dx”or “SEER Combined Mets at DX”. It records whether there is metastasis in a specific organ (such as lung, bone, liver, brain), but generally does not record the specific number of metastatic lesions within that organ. Therefore, this article cannot conduct further subgroup classification research on lung metastasis, and this defect needs to be addressed through further research in the future.

5.**The analysis is based on overall survival. Including cancer-specific survival might provide more direct insight into the effect of surgery on disease progression.**

Our response：I am extremely grateful for the valuable comments from the reviewer. I reanalyzed the data and excluded patients who did not have cancer-specific deaths. I then selected a more scientifically sound cancer-specific survival rate as the observation indicator.

6.**The abstract mentions “1,8006” cases, which appears to be a typo (likely 1,806). Please verify all numerical values throughout the manuscript for consistency.**

Our response：Thank you very much to the reviewer for pointing out the issue. This is indeed a mistake in my writing and I have corrected the numbers.

7.**The study is restricted to patients aged 60–79 years. This limits the applicability of findings to younger or older populations. Please discuss this explicitly.**

Our response：People aged 60 and above are at a higher risk of developing prostate cancer. Individuals over 79 years old generally have poorer physical conditions. Therefore, for patients aged 60 to 79 with prostate cancer and lung metastasis, it is necessary to consider the surgical treatment effect, as they can better tolerate the surgical trauma. Hence, the author chose the population aged 60 to 79 as the research subjects.

I made the following explanations in the article.：

≥ 60 years old is the high-risk group for prostate cancer. The group over 79 years old has relatively poorer physical condition. Therefore, for the age group of 60 to 79 years old, surgical treatment is necessary, and they can better withstand the trauma caused by the surgery. Therefore, the author chose the age group of 60 to 79 years old as the research subjects.

8.**The type, timing, and dosage of radiotherapy and chemotherapy are not detailed. These treatments likely influence survival and should be considered in the analysis or at least discussed as potential confounders.**

Our response：Thank you very much to the reviewer for your valuable suggestions! To reduce the interference of confounding factors such as radiotherapy and chemotherapy on the results, I conducted PSM processing on the data of the surgical group and the non-surgical group. As for the dosage and duration of radiotherapy and chemotherapy, I'm very sorry that there are no detailed records in the SEER database. Additionally, I further conducted survival analysis of the chemotherapy and radiotherapy subgroups, and the results showed that neither radiotherapy nor chemotherapy could change the survival period of the patients. As shown in the following figure:

9.**The caliper value of 0.02 is quite strict and may have excluded many patients. Justify this choice or consider using a more conventional** **caliper (e.g., 0.05–0.10) to improve matching efficiency.**

Our response：I'm very sorry. Due to a mistake in my writing, I have caused you confusion. The original caliper value in my article was 0.5, not 0.02. I have corrected this error in the article.

10.
T**he conclusion states that cytoreductive prostatectomy “does not prolong survival,” but the study only shows no statistical difference. Consider rephrasing to reflect that no survival benefit was observed in this cohort, rather than a definitive absence of benefit.**

Our response：Thank you for the very rigorous comments provided by the reviewer. I have revised the wording of the article accordingly. Additionally, I have conducted further subgroup analyses on the data in the article, which led to more in-depth conclusions.

The revisions I made in the article are as follows：

This study demonstrates that  in patients aged 60 to 79,cytoreductive prostatectomy failed to prolong cancer-specific survival in prostate cancer patients with lung metastases, nor did the combination of chemotherapy and radiotherapy extend cancer-specific survival.

**Reviewer #2: 1.Limited to a Specific Age Group: The study only included prostate cancer patients aged 60 to 79, which limits the external validity of the results (i.e., whether the results can be applied to other age groups).** **Younger or older patients may have different survival prognoses, so the study's conclusions cannot be directly applied to these groups.**

Our response：Thank you for the very rigorous comments provided by the reviewer. People aged 60 and above are at a higher risk of developing prostate cancer. Individuals over 79 years old generally have poorer physical conditions. Therefore, for patients aged 60 to 79 with prostate cancer and lung metastasis, it is necessary to consider the surgical treatment effect, as they can better tolerate the surgical trauma. Hence, the author chose the population aged 60 to 79 as the research subjects.

I have made corrections to the conclusion section of the article as follows:

This study demonstrates that  in patients aged 60 to 79,cytoreductive prostatectomy failed to prolong cancer-specific survival in prostate cancer patients with lung metastases, nor did the combination of chemotherapy and radiotherapy extend cancer-specific survival.

**2.Lack of Stratified Analysis: The absence of stratified analysis is an important limitation of the study. Stratified analysis could assess the treatment effects based on different patient characteristics (such as age, PSA levels, tumor staging, ISUP grading, etc.), helping to identify subgroups of patients who may benefit from specific treatments. Although the study used propensity score matching (PSM) to reduce selection bias, it did not conduct further stratified analysis, making it impossible to explore whether different patient subgroups have different survival benefits after undergoing prostatectomy.**

Our response：Thank you for the valuable comments from the reviewer. I further conducted subgroup survival analysis on radiotherapy and chemotherapy in the article. The data showed that radiotherapy and chemotherapy could not extend the tumor-specific survival time of the patients. As shown in the results:

**3.Lack of Multivariable Regression Analysis: The absence of multivariable regression analysis is another limitation. Including multivariable regression analysis could help further quantify the independent effects of surgical treatment and other clinical factors on the survival of prostate cancer patients, providing more detailed statistical evidence. This would not only improve the precision of the study but also offer more meaningful guidance for clinical treatment.**

Our response：Thank you for the valuable comments from the reviewer. Initially, the research plan of this author was to control confounding factors through PSM, first conducting a generation analysis for the two groups, and then further conducting regression analysis to identify risk factors. However, when I analyzed the data, there was no statistically significant difference in the survival time between the two groups, so I did not proceed with the regression analysis.

**Reviewer #3: This retrospective SEER analysis evaluates whether cytoreductive prostatectomy (cRP) confers a survival benefit in men aged 60–79 with prostate cancer and lung metastasis (2010–2015). After 1:1 PSM (79 vs 79), the Kaplan–Meier comparison shows no OS difference (log-rank P=0.79), and the manuscript concludes cRP does not prolong survival in this subgroup.** **The clinical question is relevant; however, reporting inconsistencies,** **methodological limitations (time-related bias, missingness handling, PSM details), and presentation issues currently preclude reliable inference.**

Our response：Thank you very much for your valuable suggestions on this research. We have carefully read the identified flaws and have conducted targeted verification and supplementary analysis based on the actual data of the article:

**Regarding "report inconsistency"**

We have carefully proofread the manuscript and unified the professional terms (such as "cytoreductive prostatectomy") throughout the text. There are slight differences in the description of the number of cases in the screening flowchart and the main text. This was my mistake and has been corrected in the article; the caliper value in the article is 0.7, not 0.02. I have corrected this error in the article. We have uniformly proofread and corrected the data throughout the manuscript to ensure that all case numbers, percentages, and P-values in the entire text are accurate and consistent.

**Regarding the handling of missing data:**

Due to the presence of many missing values in the database, the author used the PSM method to reduce the influence of confounding factors, and in all parameters, we ensured that there were no missing values for the parameters of survival time, survival status, and surgical treatment methods, thereby ensuring the credibility of the research results.

**Regarding the explanation of time-related bias:**

The "time-related bias" you mentioned is very important. In observational studies, ensuring the correct sequence of treatment (surgery) and outcome (death) is crucial. In this study, the cases included in the database were all prostate cancer with lung metastasis, and the SEER database recorded the clear timeline from diagnosis to the last follow-up or death. The surgery was performed after the diagnosis of lung metastasis was confirmed. To clarify this point more clearly and avoid ambiguity, we have added the following statement in the article:

"To ensure the correctness of the temporal sequence, in this analysis, all survival times are calculated from the date of diagnosis of lung metastasis, thereby avoiding the bias caused by including the time before metastasis in the survival analysis."

PSM Details:

We agree that more detailed PSM information should be provided to enhance reproducibility. We have added the following information in the "Propensity score matching (PSM)" section of the methods:

**“**After matching, the standardized mean differences (SMD) of all covariates were all less than 0.1, indicating good balance between the groups. **”**

**Reviewer #4: Nice, well designed and soundly modeled retrospective study on SEER data. Since the study is not controlled trial,** **I would recommend to authors to downplay their expression in Conclusion section when they talk about how the study confirmed non suitability of surgery in patients with lung metastasis, unfortunately this kind of studies can only suggest relationships, not confirm them.**

Our response：Thank you to the reviewer for your positive evaluation of the article and for your valuable suggestions. I have made relevant revisions to the expression in the conclusion section. As follows:

This study demonstrates that  in patients aged 60 to 79,cytoreductive prostatectomy failed to prolong cancer-specific survival in prostate cancer patients with lung metastases, nor did the combination of chemotherapy and radiotherapy extend cancer-specific survival.

6. PLOS authors have the option to publish the peer review history of their article ([what does this mean?](https://journals.plos.org/plosone/s/editorial-and-peer-review-process#loc-peer-review-history)). If published, this will include your full peer review and any attached files.


If you choose “no”, your identity will remain anonymous but your review may still be made public.


**Do you want your identity to be public for this peer review?** For information about this choice, including consent withdrawal, please see our [Privacy Policy](https://www.plos.org/privacy-policy).

Reviewer #1: **Yes:** Mohammad Ebrahimnezhad

Reviewer #2: No

Reviewer #3: **Yes:** Yuyi Ou

Reviewer #4: **Yes:** Benjamin Benzon

[NOTE: If reviewer comments were submitted as an attachment file, they will be attached to this email and accessible via the submission site. Please log into your account, locate the manuscript record, and check for the action link "View Attachments". If this link does not appear, there are no attachment files.]

While revising your submission, please upload your figure files to the Preflight Analysis and Conversion Engine (PACE) digital diagnostic tool, <https://pacev2.apexcovantage.com/>. PACE helps ensure that figures meet PLOS requirements. To use PACE, you must first register as a user. Registration is free. Then, login and navigate to the UPLOAD tab, where you will find detailed instructions on how to use the tool. If you encounter any issues or have any questions when using PACE, please email PLOS at [figures@plos.org](mailto:figures@plos.org). Please note that Supporting Information files do not need this step.

*In compliance with data protection regulations, you may request that we remove your personal registration details at any time.* [*(Remove my information/details)*](https://www.editorialmanager.com/pone/login.asp?a=r)*. Please contact the publication office if you have any questions.*
