# Supplementary material for: Survival study of cytoreductive prostatectomy for prostate cancer with lung metastasis: A propensity score matching study based on the SEER database
Source: PLoS One. 2026 Feb 23;21(2):e0342758. doi: 10.1371/journal.pone.0342758 (PMC12928561; doi:10.1371/journal.pone.0342758)
Supplement: Attachment — Submitted filename: Response_to_Reviewers_auresp_2.docx [file pone.0342758.s003.docx]

If the reviewer comments include a recommendation to cite specific previously published works, please review and evaluate these publications to determine whether they are relevant and should be cited. There is no requirement to cite these works unless the editor has indicated otherwise. 
Our response：The reviewers did not have any recommendation requirements in this regard for my article.

Please review your reference list to ensure that it is complete and correct. If you have cited papers that have been retracted, please include the rationale for doing so in the manuscript text, or remove these references and replace them with relevant current references. Any changes to the reference list should be mentioned in the rebuttal letter that accompanies your revised manuscript. If you need to cite a retracted article, indicate the article’s retracted status in the References list and also include a citation and full reference for the retraction notice.

Our response：Thank you very much, editor, for checking the article. I have checked all the reference file formats and corrected the errors.

Reviewer #4: Please rephrase the following text: "This study demonstrates that in patients aged 60 to 79,cytoreductive prostatectomy failed to prolong cancer-specific survival in prostate cancer patients with lung metastases, nor did the combination of chemotherapy and radiotherapy extend cancer-specific survival." in to something like this: "This study did not show association between cancer specific survival benefit and cytoreductive prostatectomy in 60 to 79 years old patients with lung metastases. The same can be stated for chemotherapy and radiotherapy."

Our response：Thank you for the valuable comments of the reviewers. I have revised my conclusion in the article.
